# Benefits of a Compensating Dermocosmetic Routine as an Adjunct to Treatments in Patients With Mild to Very Severe Acne: Results From an Observational Multicenter Study

**DOI:** 10.1111/jocd.70531

**Published:** 2025-11-12

**Authors:** James Odeimi, Margot Broallier, Elena Sotiriou, Monika Heizerová, Adam Reich, Dominika Kwiatkowska, Samir Salah

**Affiliations:** ^1^ La Roche‐Posay Laboratoire Dermatologique Levallois‐Perret France; ^2^ First Department of Dermatology and Venereology Aristotle University of Thessaloniki Thessaloniki Greece; ^3^ Dermatovenerologická Ambulancia Procare PentaHospitals Bratislava Slovakia; ^4^ Department of Dermatology, Institute of Medical Sciences University of Rzeszow Rzeszow Poland

**Keywords:** acne, adjunctive treatment, dermocosmetic routine, quality of life

To the Editor,

*Acne vulgaris*, irrespective of its severity, profoundly impacts patient quality of life [1, 2, 3]. While effective, topical and systemic acne medications often disrupt the skin barrier, causing dryness and irritation. These side effects, alongside acne's chronic nature and need for prolonged treatment, can significantly reduce patient adherence, ultimately compromising outcomes [3, 4, 5]. Therefore, expert consensus recommends incorporating a compensating cleanser and moisturizer into acne management to support skin barrier function, alleviate irritation, and improve tolerability. However, robust clinical evidence supporting the adjunctive use of dermocosmetics with medical therapy remains limited [4].

A multicenter observational study was conducted across five countries (Poland, Greece, Korea, Czech Republic, and Portugal) to evaluate the benefits of a compensating DC routine (cleanser and cream containing barrier‐restoring Glycerin, soothing Niacinamide, microbiome‐protecting *Aqua Posae Filiformis*, and sebo‐regulating *Bixa Orellana* seed extract) as an adjunct to local and/or systemic treatments for patients with mild to very severe acne over 3 months.

This study enrolled 2061 patients (59.3% female), aged 12–66 years, encompassing all Fitzpatrick phototypes (I–VI). At inclusion, all participants presented with mild to very severe facial acne (GEA score 2–5) and exhibited acne sequelae, including post‐inflammatory hyperpigmentation (36.5%) and erythema (66.2%). Upon enrollment, patients were primarily receiving either a local treatment (26.2%) such as adapalene or benzoyl peroxide, a systemic treatment (mainly oral isotretinoin (43%)), or a combination of both (14.9%). Patients used the DC routine at least once daily; 91.1% also applied sunscreen.

Acne severity was assessed using the Global Acne Severity (GEA) scale, ranging from 0 (clear) to 5 (very severe). Dermatologists evaluated sebum levels and improvements in skin barrier signs (erythema, desquamation, and dryness). Patients assessed skin discomfort symptoms (itching, tingling, and burning sensations), products' cosmeticity, and quality of life using the Cardiff Acne Disability Index (CADI) questionnaire. Local tolerance and satisfaction rates were rated by both dermatologists and patients at the study's end.

After 3 months of treatment, the DC routine demonstrated significant improvements in skin barrier signs, with at least a one‐grade improvement reported by 72.1% of patients for erythema, 74.7% for desquamation, and 69.7% for dryness (*p* < 0.001). Additionally, at least 82.5% of patients reported a significant reduction in skin discomfort symptoms including itching, tingling, and burning sensations (*p* < 0.001) as detailed in Figure 1. Additionally, sebum levels were significantly reduced by 55.7% (*p* < 0.001).

**FIGURE 1:**
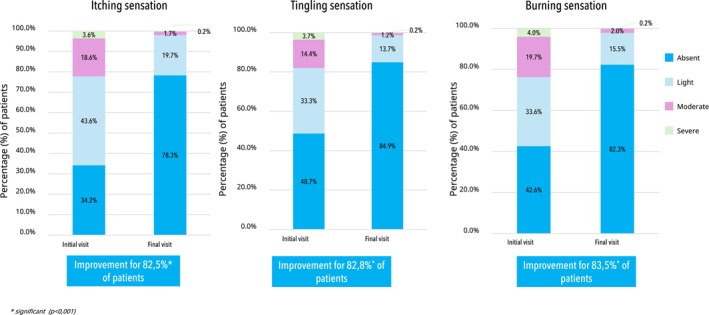
Patient‐reported improvement in skin discomfort symptoms at 3 months.

Concerning acne severity, 81.5% of patients experienced at least a one‐grade improvement in acne severity (*p* < 0.001). This clinical improvement was reflected in patients' quality of life, with the CADI score showing a significant reduction of 60.3% (*p* < 0.001) and over 72% of patients reporting at least a one‐grade improvement across all CADI items (*p* < 0.001) (Figure 2).

**FIGURE 2:**
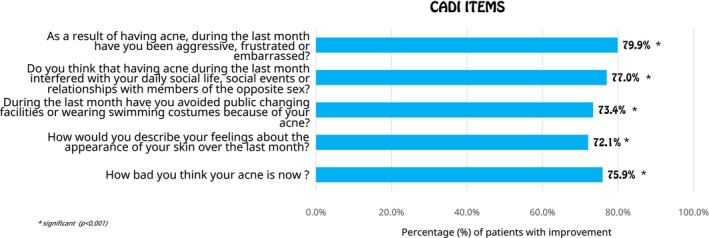
Patient‐reported improvement in quality of life (CADI questionnaire items) at 3 months.

The DC routine yielded high satisfaction and excellent tolerance rates from both patients and dermatologists (all over 95.0%). Additionally, 93.2% of patients preferred it compared to the previously used dermocosmetics regimen.

Finally, patients highly appreciated the DC routine's cosmetic attributes. The cream was reported by 94% as non‐greasy, non‐sticky, and quickly absorbed, with over 95% agreeing it left their skin feeling comfortable, softer, and hydrated. The cleanser was deemed easy to rinse and non‐drying by 95%, requiring no friction. Overall, more than 94% found the routine gentle and suitable for their skin type.

In conclusion, this study demonstrated that incorporating a compensating DC routine as an adjunct to acne medications improves tolerability and patient quality of life. These findings reinforce the positive association between adherence to acne drugs and improved outcomes in chronic conditions like acne, a common challenge for dermatologists [5]. Further research with longer follow‐up is warranted to investigate the long‐term impact on treatment adherence, overall efficacy, and relapse potential in acne patients.

## Author Contributions

Margot Broallier wrote the protocol, supervised the study globally and validated the final data. Elena Sotiriou, Monika Heizerová, Adam Reich and Dominika Kwiatkowska supervised the study locally and validated the generated data from their countries. James Odeimi, Margot Broallier and Samir Salah coordinated the post‐study activities and wrote the manuscript. All authors analyzed the data, read and approved the final manuscript.

## Consent

All subjects provided written informed consent prior to their participation in this study.

## Conflicts of Interest

Margot Broallier, James Odeimi and Samir Salah are employees of L'Oréal Group. All other authors have no conflicts of interest to disclose.

## Data Availability

The data that support the findings of this work are available from the corresponding author upon reasonable request.
